# Perceived motivators and barriers to consuming a plant-based diet: a qualitative research study

**DOI:** 10.1186/s40795-025-01100-7

**Published:** 2025-06-02

**Authors:** Jiaqi Yang, Lauren Bernard, Audrey Ting, Valerie K. Sullivan, Casey M. Rebholz

**Affiliations:** 1 Department of Epidemiology, Johns Hopkins Bloomberg School of Public Health, Baltimore, MD USA; 2 Welch Center for Prevention, Epidemiology, and Clinical Research, Johns Hopkins University, Baltimore, MD USA; 3 School of Medicine, University of Maryland, Baltimore, MD USA

**Keywords:** Qualitative research, Plant-based diet, Vegetarian diet, Vegan diet, Motivators, Barriers, Eating behavior, Environmental sustainability

## Abstract

**Background:**

Following a plant-based diet is associated with reduced risk of cardiometabolic diseases and lower greenhouse gas emissions. Yet, the determinants of plant-based diet adoption in the United States remain poorly understood.

**Objective:**

Our study aimed to identify motivators and barriers to following a plant-based diet in Baltimore-area vegan and vegetarian communities.

**Methods:**

Semi-structured individual interviews were conducted in person or via videoconference during July and August 2023. Men and women, white and non-white adult participants (*n* = 12), who were either currently consuming a plant-based diet or interested in starting to consume a plant-based diet, were recruited from Baltimore-area communities. Thematic analysis was performed using verbatim transcripts and coded with NVivo R1 (2020) (version 1.7.1).

**Results:**

Six key themes related to motivators and barriers to consuming a plant-based diet were: 1. personal factors and cultural beliefs; 2. social influences; 3. consumption of plant-based diets at home and away from home; 4. challenges in awareness and access; 5. perceptions of meat and dairy alternatives; and 6. external facilitators. Beliefs driving dietary adoption were related to animal welfare, environmental concerns, health, and culture. Influences from loved ones played an important role in shaping dietary choices. Barriers included a lack of inclusiveness, insufficient education on nutritional quality and food preparation skills, and limited availability of plant-based products. Meat and dairy alternatives were common components of plant-based diets and helped with diet transition, though nutritional quality was a concern. Facilitators included improved access to affordable, healthy foods, recipes, and support from others.

**Conclusions:**

Findings can inform strategies for promoting plant-based diets by supporting individuals to overcome social challenges, providing nutrition-related information and education, and improving access to affordable, high-quality plant-based products and meat alternatives.

**Supplementary Information:**

The online version contains supplementary material available at 10.1186/s40795-025-01100-7.

## Introduction

Plant-based diets emphasize primarily plant-derived foods while allowing for limited amounts of animal-derived products [1, 2]. Vegan, vegetarian, pescetarian, and flexitarian diets are types of plant-based diets [2, 3]. A healthy plant-based diet offers various health benefits, including lower risks of obesity, hypertension, type 2 diabetes, chronic kidney disease, cardiovascular disease, cardiovascular mortality, and all-cause mortality [1, 4–8]. Globally, plant-based diets are most prevalent in Asia (19%), followed by the Middle East and Africa (16%), Central and South America (8%), and North America (6%) [9]. Yet, the Western diet is characterized by excessive consumption of meat and overly processed foods, and only 5% of the U.S. adult population follow vegetarian or vegan diets, as of 2022 [10, 11]. Despite the known health risks associated with high consumption of red and processed meat, the perception of meat as the default protein source and its integration into many traditional American dishes sustains its high demand [12, 13].

The over-consumption of red and processed meat has strained the U.S. food system beyond its environmental limits, as high-value agricultural land is becoming increasingly scarce [14]. Compared to plant-derived foods, the large-scale agricultural production of animal products (e.g., red meat) requires substantial water and land use. On the other hand, plant-based diets have lower greenhouse gas emissions and water footprint [15]. Reliance on plant protein sources helps biodiversity and freshwater conservation as well as climate regulation, which aligns with the sustainable diet goals promoted by the Food and Agriculture Organization of the United Nations and the World Health Organization [14, 16, 17].

Previous studies exploring motivators for adopting a plant-based diet have used quantitative approaches with a predetermined list of values, whereas others have used an open-ended approach to capture a broad range of motivators and inform future interventions [18, 19]. The Theory of Planned Behavior (TPB) has been widely adopted, which includes three constructs: (1) attitude (i.e., expectations and values of outcomes associated with performing a behavior), (2) social norms (i.e., normative expectations of others and the perceived social pressure to adopt or avoid a behavior), and (3) perceived behavioral control (i.e., factors or power that facilitate or hinder adopting a behavior) [20]. To predict behavioral intention and examine underlying perceptions and beliefs of dietary habits, the TPB framework has been widely applied in diverse populations [21–23].

A qualitative study conducted in Australia identified animal welfare as the most common motivation among vegans and vegetarians, followed by environmental and health reasons [24]. Yet, the majority of the study sample consisted of female participants. There is a need for gender-balanced studies to account for the potential influence of masculinity and gender roles in reducing meat intake. Moreover, a UK study conducted in 20 young adults uncovered key differences in the motivational and psychological drivers of following a meat-free diet compared to a vegan diet, highlighting the importance of studying motivators among individuals with varying levels of animal product consumption to inform strategies for reducing animal product intake [25].

A review using evidence mapping identified health, sensory preferences, animal welfare, environmental considerations, and weight management as the most commonly reported motivations for adopting plant-based diets worldwide, including North America, Europe, Australia/New Zealand, and the Middle East [2]. In the United States, there has been a growing awareness of the health and environmental impact of one’s diet. A recent study using the International Food Information Council’s Food and Health Surveys reported that a quarter of Americans reduced their red meat consumption from 2020 to 2022 [11]. The study also noticed an increase in adherence to plant-rich dietary patterns [e.g., vegetarian, vegan, Mediterranean, flexitarian, plant-based, or Dietary Approaches to Stop Hypertension (DASH) diet], from 12 to 26%, especially among younger consumers [11, 26]. Yet, another 16% reported higher red meat consumption. The reasons for the low adoption of plant-rich dietary patterns remain unclear.

A scoping review of 24 studies revealed that most participants were in the pre-contemplative stage of dietary change and indicated a higher likelihood of adopting a diet that was similar to their own [27]. This suggests that promoting plant-based diets with reduced meat consumption may be more effective than advocating for the complete elimination of meat. However, to the best of our knowledge, no qualitative studies have explored factors that influence the consumption of plant-based diets in the American adult population using the TPB framework. As the food system and attitudes to food or portion sizes vary by region and culture, factors related to consuming plant-based diets may be unique to the U.S., where diverse ethnic groups are present, warranting further exploration [28, 29].

The present study aimed to investigate motivators and barriers to consuming a plant-based diet among U.S. community members using a qualitative approach. Identifying determinants of adherence to a plant-based diet is important for informing strategies to optimize recruitment, engagement, and adherence in future feeding trials, as well as for promoting the consumption of healthy plant-based diets in the general population.

## Methods

### Study design and participant recruitment

The present qualitative research study, Eating, Acceptability, and Taste (EAT) Plants 1, was the first of two studies with the overall goal of assessing motivators and barriers to consuming plant-based diets and acceptability of plant-based diets. In EAT Plants 1, we recruited participants living in the United States, aged between 18 and 65 years, who were able to speak, read, and understand English, had access to the internet if participating online, and were either currently consuming a plant-based diet or interested in starting to consume a plant-based diet.

Our study targeted a sample size of 12 individuals to largely achieve theoretical saturation and to not exceed financial constraints. This estimation assumed an over 85% probability of identifying a theme if it was held by at least 15% of the target population [30]. The final obtained sample size (*N* = 12) achieved data saturation as no new sub-themes emerged and common themes were consistently identified across participants. Furthermore, we aimed to recruit a balanced sample of men and women as well as a racially diverse group of participants from Baltimore-area vegetarian and vegan communities. Participants were recruited by posting study flyers on social media platforms (e.g., Facebook groups such as Baltimore Vegans, Vegan Maryland, DMV Vegans; Twitter/X via Johns Hopkins Welch Center @JHUWelchCenter; and the Johns Hopkins Welch Center weekly e-newsletter) as well as posting flyers across the Johns Hopkins medical campus (e.g., Bloomberg School of Public Health, School of Nursing, the Welch Center) and Northeast Market to encourage participation from community members broadly. Additionally, during in-person promotion at local events (e.g., Johns Hopkins farmers market), flyers and cards with study descriptions were distributed.

Eligible participants were invited to attend a one-time, 60-minute interview, conducted in person at the Johns Hopkins Welch Center for Prevention, Epidemiology, and Clinical Research in Baltimore, Maryland, or virtually via Zoom, depending on participants’ availability and preference. Participants received a $50 gift card (Visa or Amazon) as compensation for their time upon interview completion.

### Procedure and materials

The study protocol was reviewed and approved by the Institutional Review Board Office at Johns Hopkins Bloomberg School of Public Health (IRB00025358). Recruitment materials, including flyers and cards, were designed with the study’s email address and phone number, allowing prospective participants to contact the research team directly. Trained study team investigators (J.Y., A.T.) conducted screening to identify eligible participants and obtained oral consent over the phone. Individual interviews took place between July and August 2023, and no eligible participants refused enrollment or dropped out. A semi-structured interview guide was developed based on the TPB (**Appendix**), and two moderators (C.M.R., V.K.S.) facilitated the interview on motivators and barriers to adopting a plant-based diet [31–33]. The interview questions were open-ended and based on three constructs: attitude, social norms, and perceived behavioral control [31, 32]. We also incorporated price-related topics to address potential equity issues. The interviews were completed after all questions were asked and answered or after one hour had elapsed, whichever occurred first. The moderator (C.M.R.) made field notes during the interviews. Afterward, demographic data (age, gender identity, race/ethnicity) of participants were collected over the phone and securely stored in REDCap for subsequent analysis. All interview sessions were audio recorded.

### Coding and analysis

The audio recordings were stored as m4a files to use for the qualitative analysis. To ensure participant confidentiality, two investigators (J.Y., A.T.) from the study team de-identified the audio transcripts by assigning unique study IDs (e.g., “01”). Transcriptions were automatically generated via Zoom. To ensure the accuracy of transcripts, two investigators (J.Y., A.T.) verified transcripts verbatim by listening to audio recordings multiple times. NVivo R1 (2020) (version 1.7.1 Denver, Colorado), a qualitative data analysis computer software, was used for thematic analysis.

Data were analyzed following the reflexive approach to thematic analysis. Developed by Braun and Clarke, reflexive thematic analysis emphasizes the researcher’s active role in identifying and interpreting patterns of meaning across the interviews [34, 35]. We adopted a six-phase analytical process: (1) data familiarization, (2) generating initial codes, (3) developing themes, (4) reviewing themes, (5) defining and naming themes, and (6) reporting findings [35]. First, two investigators (J.Y., L.B.) familiar with the transcripts took preliminary notes to inform the thematic framework. Two of the 12 transcripts were coded in duplicate by two investigators (J.Y., L.B.) independently for quality assurance. Then, two investigators (J.Y., L.B.) compared and selected codes that were conducive to interpreting themes and discussed with two additional investigators (C.M.R., V.K.S.) from the team to refine the codes. One investigator (J.Y.) applied the codes to the remaining transcripts and collated codes into initial candidate themes and sub-themes. Finally, the team (C.M.R., V.K.S., L.B., J.Y.) reviewed candidate themes, examined the quality and boundaries of each theme, and defined and named each theme.

## Results

The study involved 12 adult participants (7 women and 5 men), with a median age of 32 years (range, 21–62 years), and 9 participants (75%) were White (Table 1). Most participants were vegan or vegetarian. Two consumed limited amounts of animal flesh foods a few times per month or seasonally (flexitarian). The participants had been following a plant-based dietary pattern for a long duration, ranging from 2 to 38 years, with a median of 6 years.

**Table 1:** Demographic characteristics of EAT plants 1 participants

Variable	Statistic
*N*	12
Age (years)	
Median (25th, 75th percentile)	32 (24.5, 51.5)
Minimum, maximum	21, 62
Female, *n* (%)	7 (58)
Racial group, *n* (%)	
White	9 (75)
Asian American or Pacific Islander	2 (17)
Other (half Asian, half White)	1 (8)
Current diet, *n* (%)	
Vegan	7 (58)
Vegetarian	3 (25)
Flexitarian	2 (17)

From the thematic analysis, we identified six key themes related to motivators and barriers to consuming a plant-based diet: [1] personal factors and cultural beliefs; [2] social influences; [3] consumption of plant-based diets at home and away from home; [4] challenges in awareness and access; [5] perceptions of meat and dairy alternatives; and [6] external facilitators. Each key theme was presented individually, along with sub-themes and representative quotations used to illustrate central points (Table 2).

**Table 2:** Themes, sub-themes, and representative quotes derived from the interviews conducted in 12 study participants

Theme	Sub-theme	Representative quote
1. Personal factors and cultural beliefs	1.1 Ethics and animal rights	“And now I care less about the health, the reason for myself, and it’s more for ethical reasons.” (ID 05) “And then I started volunteering in an animal sanctuary, and I had, like a closer relationship to like cows and pigs and things like that. So I just felt like I was like disrespectful to animals.” (ID 12) “The initial change was for sort of moral, compassionate reasons.” (ID 07) “I know deep down in my heart, that it’s not right what we’re doing to other creatures. I also think for most vegans. That’s the way to sustain it is just having it as a bedrock value that it’s not right.” (ID 04) “The most important reason I consume the diet is for ethical reasons.” (ID 08)
	1.2 Health effects	“I did it mostly for my health. A number of years ago, when I got ulcerative colitis, I was looking into different diets, and I realized that a plant-based diet, if done properly, could make the inflammation go down. And so I started on a plant-based diet. And it worked!” (ID 01) “I was told I had like a dairy sensitivity, so I removed it from my diet and then I gradually kind of went plant-based after that.” (ID 05) “I’ve been anemic before, which is just common among reproductive-age women [who] menstruate. So, I guess feeling like, maybe, you know, there’s no way to probably overcome that from a dietary perspective…maybe not getting all the nutrients I could get from, you know, non-plant-based sources.” (ID 06)
	1.3 Culture and religion	“Cultural reason for me, as an Indian, the South Indian vegetarian, I mean, I never grew up with meat in my house…I can say my parents, to an extent, disapprove of me not eating a plant-based diet for religious and cultural reasons, but as I’ve gotten older, I also care less about what my parents say, so it doesn’t really matter. It’s kind of what I want.” (ID 02) “I’m vegetarian, and I have been since I was born because my family just raised me that way.” (ID 03)
	1.4 Environment and climate change	“And for the environmental health as already proven. Then that’s why I’ve stuck with it… For like water use is a very big thing because maintaining farms and places where meat is processed uses a ton a ton a ton of water.” (ID 03) “Back, you know, years ago when I started, maybe… it’s a little bit more now because of the seeming urgency of climate change.” (ID 11)
	1.5 Weight management	“I feel like it’s easier to keep my weight down. You know, even though I’m now post-menopausal, do gain a little weight now and then. And I feel like it’s a good way just to, to stay healthy.” (ID 04) “I feel like adhering to a plant-based diet does make it easier to have a long-term weight management goal if I want to stay as fit, partially because of the satiety of plant-based foods. Especially if I truly have a portion of my meal that’s like whole vegetables and whole grains, so there’s only so much you can eat, plus the fiber content also makes it difficult to digest everything I’m eating.” (ID 02)
2. Social influences	2.1 Influence from loved ones	“For the approving side, I would definitely say my family. And I think that’s just because they also follow a plant-based diet. It also aligns with, you know, cultural values that they held when they first raised me that way. So in that way they’re pretty approving. My significant other also eats a plant-based diet.” (ID 03) “I’m probably a little less plant-based now, just because my partner was vegan for most of our relationship, and he has just recently decided he doesn’t want to be a strictly vegan.” (ID 06)
	2.2 Influence from peers	“One of my friends would not be happy with me if I walked to an Indian buffet and just get the dessert and like did not touch the raw vegetables. So, like dessert and rice, and really just fatty curry that they have, you know. ‘But you have to eat your vegetables’… At first, it’s a joke, but that’s the real pressure.” (ID 02) “I grew up in a very conservative town…I had a friend who was vegan in high school, and I saw the way people treated her, including myself - like I’ll be totally honest, like just the judgment that she experienced, and I was like ‘I could never do that,’ just seeing the way she was treated for it. So that’s just- that was the norm to me, but I don’t experience it now.” (ID 09) “Also, the social aspect. You know, there’s sort of a stigma around veganism that I didn’t really want to be a part of…I didn’t wanna experience like the bullying that comes with being a vegan.” (ID 09)
	2.3 Influence from authoritative figures	“[One] person I need to add to that list is my primary care doc. I’ve only had a handful conversations for my primary care. Remember the whole message for me is saying, should consider eating healthier if you want to gain weight. There’re ways you can do that, without just eating sugar. Here’s a list of plant-based foods that are higher in like good fats and higher calories by definition.” (ID 02) “I’m influenced by opinions of experts in the field… one major one is like, who’s done major nutrition research is Walter Willett…There was one paper that came out on eggs that he did, and my parents ended up consuming a lot fewer eggs.” (ID 10)
	2.4 Influence from books, classes, and social media	“I did read books about like environmental impact. So, I mean, that just kind of like strengthened my resolve.” (ID 12) “I took an interesting class like a few years ago. I’ve been eating more vegetables since then.” (ID 12) “I think on social media, I am a lot and influenced positively, like I follow a lot of people like, let’s say, just like on Instagram, or read a bunch of articles about people who also consume plant-based [diets].” (ID 03)
3. Consumption of plant-based diets at home and away from home	3.1 Limited options when eating out and traveling	“The fact that restaurants may give you one choice, or you’re basically stuck with salad.” (ID 01) “Availability - I think one concern I have is where I go next… will I live in a place that has enough around me for me to consume? Like enough restaurants that cater to plant-based diets.” (ID 03) “I feel that I don’t have amazing access to eating a lot of the cultural foods that I did growing up…there are just not vegan versions of them, and if there are, like, they’re not nearly as accessible.” (ID 08) “Sometimes when traveling it can be hard like, I think a couple of years ago we went to Mississippi for vacation, and it was a bit of a challenge. We cooked a lot.” (ID 07) “We just got back from a road trip. Oh, I wish there was some vegan fast food out there, you know, like, it’s not healthy. But sometimes you just need to go in for something quick to eat.” (ID 04)
	3.2 Social isolation	“Right now, it seems like my social group now really likes to go out to different restaurants and try, you know, whatever type of cuisine, so I wouldn’t want to not be able to participate because of my dietary restrictions.” (ID 06)
	3.3 Ability to prepare plant-based meals at home	“It’s easier to consume a plant-based diet as it’s faster. It doesn’t involve, maybe a lot of preparation in terms of cooking. So, for example, if I just wanna put together some like rice with some oil and vegetables that can be like you can assemble that pretty quickly.” (ID 06) “I’m about, I would say, 80% plant based…I eat [at] home. When, when I’m doing well, I eat [at] home, I would say, 95% of the time.” (ID 01) “Disadvantages are more time to prep and cook because a can of beans doesn’t taste as good as a piece of fish, and [bean is] just plain, you know.” (ID 10)
	3.4 Difficulties in preparing different diets in the same household	“So, for cooking together, it’s often a little bit difficult. My partner yesterday, for example, made some vegetable stir fry along with some chicken. She made the vegetables very separately, but I want cooked vegetables separately to begin with…because I won’t just like season them on a pan with nothing.” (ID 02)
4. Challenges in awareness and access	4.1 Lack of inclusiveness and understanding	“There [are] a few things we can order, and I like those things, but sometimes you have to ask them to tailor it, and they like don’t understand. There’s also like cultural and linguistic barrier that just makes explaining too annoying or atypical.” (ID 08) “It almost is like, well, if you don’t want the meat-based option, you’re some weird, special person who’s gonna ask for additional things and need to be accommodated.” (ID 06) “Sometimes there’s a kind of mild disappointment… I had several people trying to sell me to eat sushi in Japan…and then I’m forced to explain what my opposition is being plant-based…I kind of don’t want to box myself into that position [and] have to explain my life choice to everyone all the time.” (ID 02)
	4.2 Lack of education and information	“When we grow up…we don’t learn how to grocery shop or cook food, I feel like there is not meaningful thought or consideration given to how do we prepare, consume, purchase food as individuals in society… I think also just lack of education and like information about our relationship with food.” (ID 08) “Sometimes people who attempt to go plant-based or try to go plant-based don’t really have a super good grasp on the nutrition part of it.” (ID 05) “It’s not done as seriously. There are definitely calorie labels, for sure, but I feel like there should be a more of a macro breakdown as well. And proteins, fats and carbohydrates, because a lot of the time the plant protein is quite lacking compared to the chicken or something that they put in a meal.” (ID 02)
	4.3 Lack of availability of quality vegan protein sources in restaurants or grocery stores	“And especially if I’m eating outside, it is hard to get, like a quality protein source.” (ID 02) “Maybe other concerns, sometimes it’s hard to find vegan products in certain grocery stores that [are] better.” (ID 04) “But I think in the grocery store… like all the plant-based meat products or tofu, or something, is at the bottom, and there’s like a lot smaller selection… it’s not like featured to my eye in the grocery store.” (ID 08) “It’s very hard to be vegan if you want to eat out, way harder than being vegetarian…I just didn’t eat enough of like the tofu overall to get enough protein…I knew that I could do it with beans and legumes and grains, but I didn’t feel satiated, I guess.” (ID 10)
5. Perceptions on meat and dairy alternatives	5.1 Nutritional quality of alternatives	“I don’t eat meat substitutes regularly. I try to stay away from processed foods just generally. So I try to limit my meat substitute intake.” (ID 05) “They’re high in sodium - I try to avoid like super high sodium products and high fat. So, if they were a little bit more whole, I’d probably use them more often.” (ID 09) “I guess my partner really likes all the chicken alternatives. I grew up in a household [where] my parents really would always go for those types of things. But I also can view that as pretty processed, and probably has a lot of other things added to it.” (ID 06) “I drink primarily like oat and soy milk are probably my top ones, and I do use nondairy cheeses. Not a lot. I don’t really like to [eat them] because I feel like they don’t have much of a nutritional benefit, but I’ll do if I think it’ll add something.” (ID 09)
	5.2 A way to transition to plant-based diets	“When I first became vegan, I consumed a lot more of those because it was like, oh, this is like cool product. This is similar to something that I used to eat.” (ID 08) “I think that those alternatives…are a good thing for promoting a vegan diet, because it gets people to actually enjoy the food instead of just eating mush.” (ID 12)
	5.3 Taste and price	“It’s been really popular to consume dairy alternatives now for a long time…The alternatives to cheese…melt very weird, so I would go along with buying them if someone else really wants to.” (ID 06) “I don’t really consume them as much anymore, mostly for health reasons, and because they’re more expensive.” (ID 08) “It would be nice if the meat substitutes were cheaper.” (ID 05) “There’s a lot of like rapid improvement in making vegan food taste better…there’s a lot of really good vegan cheese options, but you have to know which ones they are.” (ID 12)
6. External facilitators	6.1 Healthy, cheap, and convenient options	“The accessibility of good, cheap, healthy foods is very lacking in the United States. But in the US, I get fries and burgers, or something like that, it’s hard to get good cheap options.” (ID 02) “I think if there were like very cheap, dense- protein options that would help…Some of the vitamins, if they would put them in more foods…the vitamins that vegans don’t always get.” (ID 12) “And just convenience. I think more restaurants that are like vegetarian-heavy would be really nice…to be able to have options when you aren’t able to cook, that would be more plant-based, that’s pretty rare at restaurants.” (ID 10)
	6.2 More recipes	“I probably need to try different recipes or ways of making a number of ways to make it, you know, more palatable, but I feel like the palatability of the food is quite difficult.” (ID 02) “In general, having recipes where I would waste less food. Because when I’m at my place, it’s just, I’m making food for one person, so then it becomes easy for food to go bad.” (ID 06)
	6.3 Support from other people	“I noticed that some of the people around me who have more hostility to even trying plant-based food…I guess about why that is because they just don’t know anyone in their life who is vegan or plant-based, and not just to share the benefits of it, but like to know how to do it…I think they just don’t know how to even get started.” (ID 08) “I think having people to do it with is always great. And so, working with my mom, we’ve always kind of enjoyed coming up with new recipes trying new recipes. We add them to her cooking book…So it makes it less burdensome and just more fun.” (ID 10)

### Personal factors and cultural beliefs

This theme included reasons that participants started and continued to consume a plant-based diet. Moral compassion for animals was a major reason for consuming a plant-based diet reported by participants. Experience can shape participants’ perspectives, as one participant mentioned that volunteering in an animal sanctuary changed their feelings towards animals and their choice of diet. Some participants who consumed some animal products as part of their plant-based diet expressed some degree of struggle in dealing with their feelings towards animal welfare.

“And then I started volunteering in an animal sanctuary, and I had, like a closer relationship to like cows and pigs and things like that. So I just felt like I was like disrespectful to animals.” (ID 12)

*“*For animal welfare, I still struggle with this one because I do consume animal products.” (ID 10).

All participants were aware of the health benefits of following a healthy plant-based diet, such as improved gastrointestinal health and lower chronic inflammation. Two participants identified personal health conditions as the primary motivators for adopting a plant-based diet.

“I did it mostly for my health. A number of years ago, when I got ulcerative colitis, I was looking into different diets, and I realized that a plant-based diet, if done properly, could make the inflammation go down. And so I started on a plant-based diet. And it worked!” (ID 01).

Yet, some participants also expressed health concerns about nutritional deficiencies related to plant-based diets, particularly vitamin B_12_, and the perceived need to take supplements.

“I’ve been anemic before, which is just common among reproductive-age women [who] menstruate. So, I guess feeling like, maybe, you know, there’s no way to probably overcome that from a dietary perspective…maybe not getting all the nutrients I could get from, you know, non-plant-based sources.” (ID 06).

Two participants explained that they had followed a plant-based diet since birth due to the cultural values and religious beliefs of their families. Yet, one participant noted that the influence of their family on their dietary choices diminished as they grew older, and their reasons for adhering to the diet evolved.

“Cultural reason for me, as an Indian, the South Indian vegetarian, I mean, I never grew up with meat in my house…I can say my parents, to an extent, disapprove of me not eating a plant-based diet for religious and cultural reasons, but as I’ve gotten older, I also care less about what my parents say, so it doesn’t really matter. It’s kind of what I want.” (ID 02).

Most individuals recognized the environmental benefits of following a plant-based diet, including less water usage and greenhouse gas emissions. One participant stated the urgency of climate change as the main reason to adopt a plant-based diet.

“Back, you know, years ago when I started, maybe… it’s a little bit more now because of the seeming urgency of climate change.” (ID 11).

Lastly, none of the participants identified weight management as a main driver for consuming a plant-based diet, but they described weight control as one of the benefits of adhering to the diet. Participants’ perceptions of weight control were often tied to personal feelings of satiation during a meal, leading to less intake.

“I feel like adhering to a plant-based diet does make it easier to have a long-term weight management goal if I want to stay as fit, partially because of the satiety of plant-based foods. Especially if I truly have a portion of my meal that’s like whole vegetables and whole grains, so there’s only so much you can eat, plus the fiber content also makes it difficult to digest everything I’m eating.” (ID 02).

### Social influences

This theme described the approval and disapproval participants received when consuming a plant-based diet. Influence from loved ones, including family members and partners, was commonly reported by participants. Family culture and values of consuming a plant-based diet strongly shaped participants’ dietary habits. Sharing recipes and cooking together were ways families, whether plant-based or not, showed approval of participants’ plant-based diets. Partners’ diets also played an important role in participants’ diets.

“For the approving side, I would definitely say my family. And I think that’s just because they also follow a plant-based diet. It also aligns with, you know, cultural values that they held when they first raised me that way. So in that way they’re pretty approving. My significant other also eats a plant-based diet.” (ID 03).

Another influence participants perceived was peer pressure. They explained that this pressure encouraged them to consume a healthier plant-based diet. However, peer pressure was also negative and manifested as judgment or disapproval of a plant-based diet.

“I grew up in a very conservative town…I had a friend who was vegan in high school, and I saw the way people treated her, including myself - like I’ll be totally honest, like just the judgment that she experienced, and I was like ‘I could never do that,’ just seeing the way she was treated for it. So that’s just- that was the norm to me, but I don’t experience it now.” (ID 09).

Other perceived influences came from authoritative figures, such as physicians and nutrition researchers, who impacted their food choices. Lastly, participants reported other sources of information (e.g., books, classes, and social media) that motivated them to continue to consume a plant-based diet.

### Consumption of plant-based diets at home and away from home

This theme included factors that influence participants’ practice of consuming plant-based diets at home and away from home. When accessing foods outside the home, participants reported difficulties in finding plant-based options due to lack of availability, especially when traveling.

“Sometimes when traveling it can be hard like, I think a couple of years ago we went to Mississippi for vacation, and it was a bit of a challenge. We cooked a lot.” (ID 07).

“And especially if I’m eating outside, it is hard to get, like a quality protein source.” (ID 02).

In addition, participants frequently mentioned social pressure when eating out. In particular, they described feeling isolated due to their dietary restrictions, which often prevented them from trying different restaurants with their social group.

“Right now, it seems like my social group now really likes to go out to different restaurants and try, you know, whatever type of cuisine, so I wouldn’t want to not be able to participate because of my dietary restrictions.” (ID 06).

When preparing plant-based meals at home, participants generally reported that it was easy since it did not involve much preparation, except one participant noted the extra time and effort required for plant-based cooking compared to meat-based meals. When cooking with a partner or preparing meals for the household, participants expressed concerns if family members had different diets.

“Disadvantages are more time to prep and cook because a can of beans doesn’t taste as good as a piece of fish, and [bean is] just plain, you know.” (ID 10).

“So, for cooking together, it’s often a little bit difficult. My partner yesterday, for example, made some vegetable stir fry along with some chicken. She made the vegetables very separately, but I want cooked vegetables separately to begin with…because I won’t just like season them on a pan with nothing.” (ID 02).

### Challenges in awareness and access

This theme pointed to perceived barriers and concerns about consuming a plant-based diet. Many participants highlighted the lack of inclusiveness and understanding for people consuming a plant-based diet as a major barrier. This lack of inclusiveness was evident in the absence of default plant-based options when ordering meals in groups. Participants also reported feeling a lack of understanding from others, including difficulties in asking for tailored foods when eating out and the challenge of having to explain their dietary choices.

“Sometimes there’s a kind of mild disappointment… I had several people trying to sell me to eat sushi in Japan…and then I’m forced to explain what my opposition is being plant-based…I kind of don’t want to box myself into that position [and] have to explain my life choice to everyone all the time.” (ID 02).

Additionally, participants expressed there was a lack of education about food preparation and nutrition. For example, one participant noted a lack of meaningful consideration given to how individuals prepare, consume, and purchase food. They also highlighted a need for education on the nutrition aspects of a plant-based diet and detailed information on nutrition fact labels of plant-based products.

“When we grow up…we don’t learn how to grocery shop or cook food, I feel like there is not meaningful thought or consideration given to how do we prepare, consume, purchase food as individuals in society… I think also just lack of education and like information about our relationship with food.” (ID 08).

As mentioned by most participants, a lack of availability of good quality vegan protein options in restaurants and grocery stores was another significant barrier to consuming a plant-based diet.

“And especially if I’m eating outside, it is hard to get, like a quality protein source.” (ID 02).

### Perceptions on meat and dairy alternatives

This theme described participants’ perceptions of meat and dairy alternatives. Most participants mentioned that they consumed meat and dairy alternatives, but they were concerned about the nutritional quality of these alternatives. Several participants perceived these products as being highly processed and high in sodium, so they did not consider these alternatives to be the healthiest option for a plant-based diet.

“I guess my partner really likes all the chicken alternatives. I grew up in a household [where] my parents really would always go for those types of things. But I also can view that as pretty processed, and probably has a lot of other things added to it.” (ID 06).

Participants who were former meat-eaters often reported a preference for meat alternatives. They perceived meat alternatives as a helpful transition to a plant-based diet and a good way to promote a vegan diet to the public.

“When I first became vegan, I consumed a lot more of those because it was like, oh, this is like cool product. This is similar to something that I used to eat.” (ID 08).

Other concerns included the taste and price of alternatives. One participant reported that certain products (e.g., cheese alternatives) had weird tastes or high prices, so they did not consume them very regularly.

“It’s been really popular to consume dairy alternatives now for a long time…The alternatives to cheese…melt very weird, so I would go along with buying them if someone else really wants to.” (ID 06).

### External facilitators

This theme summarized perceived facilitators and identified potential future opportunities to make it easier to consume a plant-based diet. Several participants reported that greater accessibility to affordable and healthy foods in the U.S. would make consuming plant-based foods more convenient. Protein-dense and vitamin-enriched plant-based products were favored by some participants.

“And just convenience. I think more restaurants that are like vegetarian-heavy would be really nice…to be able to have options when you aren’t able to cook, that would be more plant-based, that’s pretty rare at restaurants.” (ID 10*)*.

Another perceived facilitator was having access to more plant-based recipes. To enhance palatability and reduce food waste, many participants expressed a desire for good recipes when cooking at home.

“In general, having recipes where I would waste less food. Because when I’m at my place, it’s just, I’m making food for one person, so then it becomes easy for food to go bad.” (ID 06).

Several participants mentioned that having people around who were also following and knowledgeable about the benefits of plant-based diets would be very helpful when getting started. This support would make the transition to a plant-based diet less burdensome.

“I noticed that some of the people around me who have more hostility to even trying plant-based food…I guess about why that is because they just don’t know anyone in their life who is vegan or plant-based, and not just to share the benefits of it, but like to know how to do it…I think they just don’t know how to even get started.” (ID 08).

### Advantages and disadvantages

**Fig. 1:**
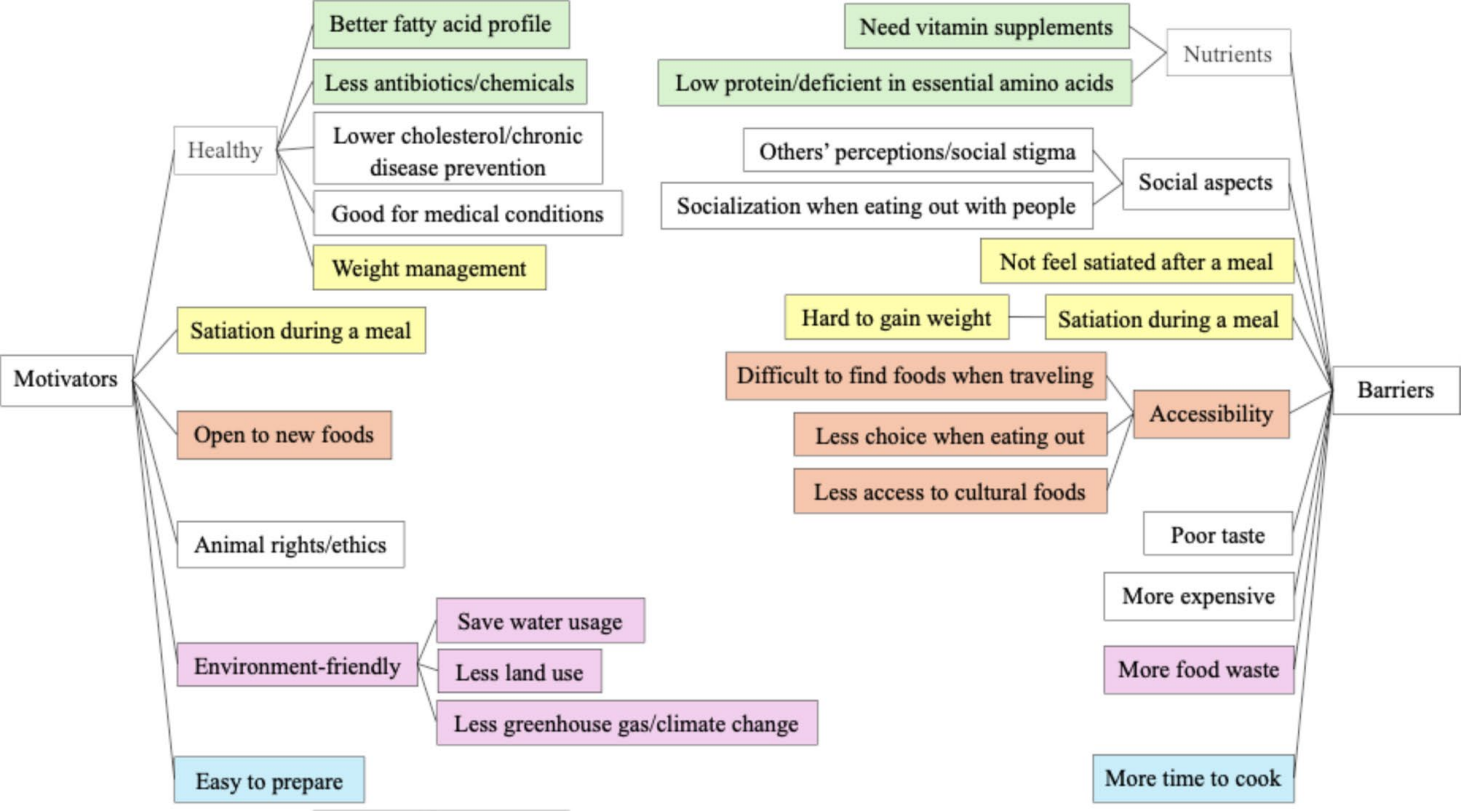
Concept map depicting motivators and barriers to consuming a plant-based diet from 12 adult participants^1^,^2^

We depicted the perceived advantages and disadvantages of following a plant-based diet as reported by participants in the concept map (Fig. 1). There were several instances of overlap with themes, and we used color shading to group phrases with similar semantics and contrasting perceptions held by different participants.

All participants believed that following a plant-based diet while limiting meat intake was beneficial for their health. Perceived health benefits included better fatty acid profiles and lower levels of antibiotics and chemicals. Yet, five participants also reported that plant-based foods often lacked certain vitamins and essential amino acids. Two participants appreciated that the plant-based diet increased their exposure to new foods, while five participants perceived a lack of access to quality plant-based options when eating out and traveling to be a significant drawback. Social discomfort was another perceived disadvantage. At home, two participants found it easier to prepare plant-based meals compared to meat-based meals, while one reported that meal preparation required considerable time and effort. Eleven participants highlighted the environmental benefits of a plant-based diet, such as conserving water and land, and reducing greenhouse gas emissions, though one was concerned about increased food waste due to spoilage of plant-based foods. Animal rights and ethics were also perceived as significant advantages. While nine participants found the plant-based diet beneficial for weight control due to feelings of fullness during ingestion, it was less beneficial for muscle-building purposes, as one participant found it difficult to gain weight. Additionally, half of the participants noted that certain plant-based products were expensive, less palatable, and left one participant feeling unsatisfied and lacking satiety after meals due to inadequate portions or insufficient protein content when eating out.

## Discussion

In this qualitative study, we explored the perceived motivators and barriers to consuming a plant-based diet among twelve Baltimore-area community members and identified six key themes. Beliefs driving the adoption of plant-based diets included concerns for animal welfare, environment, health, and cultural factors. Influence from loved ones, peers, authoritative figures, and social media also played an essential role in shaping participants’ dietary choices. Participants described that their experiences cooking at home and eating out impacted their ability to maintain a plant-based diet. Perceived barriers included a lack of inclusiveness, insufficient information on food preparation and nutritional quality, and limited availability of high-quality, affordable plant-based products. Participants reported consuming meat and dairy alternatives, despite concerns about their nutritional quality and taste. External facilitators to maintaining a plant-based diet included greater access to affordable and healthy foods, recipes, and support from others. These findings provide valuable insights for optimizing recruitment and engagement in future feeding trials and could inform realistic strategies to promote the consumption of healthy plant-based diets in the general population.

For personal beliefs that influence consuming a plant-based diet, some participants expressed strong convictions regarding ethics and animal rights as primary motivators. Previous studies have highlighted that while adopting a vegan diet can be challenging due to initial barriers such as extreme hunger, stress, and anxiety, ethical benefits were most strongly perceived by those in the contemplation/preparation and action/maintenance stages [36–38]. In line with these findings, several participants in our study who had followed plant-based diets for a shorter duration (less than the median) described ethics as the primary driver for adopting a plant-based diet. While our sample size limited broad generalizability, this pattern suggested a potential link between the stage of dietary transition and underlying motivations. For participants who included small portions of animal products in their plant-based diets, they had moral concerns about consuming animal products but found it difficult to exclude these foods entirely, which could be reflective of underlying beliefs in human supremacy over other animals [25]. Beyond ethics, participants cited environmental benefits (e.g., saving water, less land use) of consuming a plant-based diet, identifying sustainability as an important motivator considering the rising environmental burden of meat consumption. In our study, most participants cited the health benefits of plant-based diets as reasons to consume a plant-based diet. These beliefs were strongly valued by individuals with specific medical conditions, as they experienced improved health through plant-based diet adherence. Notably, these beliefs aligned with existing evidence that plant-based diets help improve inflammation and manage symptoms of certain chronic conditions [39]. In addition, many participants viewed weight management as a long-term health benefit of maintaining the diet due to satiation. Appetite during a meal is associated with satiation, a series of processes including physiological mechanisms that promote meal termination; therefore, satiation plays a role in controlling meal size [40].

Besides personal beliefs, some vegans and vegetarians were strongly influenced by socio-cultural factors, particularly the attitudes of loved ones toward plant-based diets. Family culture plays a significant role, as parents typically prepare food for the household, which shapes food preferences of young individuals [41]. However, our findings indicated that this familial influence diminished as people gained independence in adulthood. Additionally, peer pressure plays a central role in shaping individual dietary habits. Participants reported feeling pressured to adopt a healthier plant-based diet to align with their peers, which reflected the impact of social modeling of eating [42]. Other sources of influence, such as books and social media, can further strengthen people’s resolve to follow a plant-based diet. Increased exposure to dietary information from social media has been positively associated with individual perceived food norms about what foods people typically eat or should eat [43]. A previous study also identified social media exposure to food messaging as a useful tool in facilitating the consumption of a plant-based diet [44].

When eating outside the home, the availability of plant-based products was often perceived as a barrier, which is consistent with previous studies [21, 45]. Participants emphasized that while accessing plant-based products was less of a concern than it once was, availability still largely depended on where they lived. This inconvenience was particularly salient during traveling. Price was another challenge mentioned by participants, consistent with findings from a prior qualitative study conducted among younger individuals, which identified perceived cost as an important barrier to adopting a plant-based diet [44].

Moreover, participants reported experiencing a lack of social acceptance and support. Similar to previous studies, social isolation and lack of understanding were perceived as important barriers to maintaining a plant-based diet [21]. Participants reported feeling excluded when eating out with omnivores, asking for tailored foods, or having to explain their dietary choices. The lack of inclusiveness and understanding from society appears to arise from the perceived violation of dietary norms and the stigma of not consuming animal products, which is sometimes viewed as a sign of weakness [46, 47]. However, previous research has illustrated that communal eating facilitates social bonding, and socializing with others who follow a plant-based diet can provide a sense of belonging [21]. As plant-based diets become more popular, food norms are expected to change, allowing for greater inclusivity for people who consume plant-based diets. Further efforts are needed to encourage social acceptance to promote plant-based diets in the general population.

The lack of education and information was perceived as another challenge in adopting plant-based diets. Participants noted that, when starting a plant-based diet, they often lacked knowledge of grocery shopping and food preparation. Moreover, there were differing opinions on the time and effort required to prepare plant-based meals. While some participants found meal preparation simple, others described it as time-consuming. This variation may be linked to how long participants had been consuming plant-based diets, their cooking skill level, interest in food shopping and preparation, and whether they were primarily responsible for meal planning [48]. Additionally, individual relationships with foods, such as how we prepare, consume, and purchase it, often receive insufficient attention. This gap can be traced back to childhood when nutrition education was lacking. Providing nutrition education to adolescents is important for promoting healthy eating habits and improving nutritional knowledge [49]. Evidence suggests that such education can increase vegetable consumption and predict continued healthy eating in later years [50]. Moreover, detailed nutrient labeling on plant-based products when eating out can help individuals find the right plant-based options that meet their nutrient needs.

Improving the nutritional quality, palatability, and affordability of meat and dairy alternatives could be beneficial for promoting plant-based diets. Consistent with previous studies, our results suggested that most participants regularly consumed meat or dairy substitutes, indicating that these alternatives were commonly included in American plant-based diets [51]. Participants felt that having access to these substitutes made the transition to a plant-based diet easier, as these products allowed them to enjoy familiar foods they used to eat. A qualitative study conducted in Australia identified taste and enjoyment as the highest motivating factor for omnivores [24]. Moreover, previous research suggested that plant-based meat alternatives were either superior or equal to traditional meat-based products in maintaining post-meal satiety [52–54]. As such, promoting meat alternatives could be an efficient strategy to reduce the over-consumption of meat.

Participants in our study also expressed concerns about the nutritional quality of these alternatives, as many meat substitutes are highly processed to mimic the sensory properties of real meat. These substitutes sometimes contain high levels of carbohydrates, which are used to modify texture, and tend to be higher in sodium, which acts as a flavor enhancer and helps extend shelf life [55, 56]. In addition, these products are frequently fortified with micronutrients such as vitamins B_12_ and D, iron, and zinc to compensate for deficiencies in plant-based ingredients [57]. Evidence from clinical trials remains mixed regarding the effects of plant-based meat substitutes on cardiovascular disease risk factors when compared to animal-based meat products [58, 59]. Given the need for meat and dairy alternatives to reduce the over-consumption of animal products and the current uncertainty about their health impacts, further research is needed to explore the long-term health impacts of these substitutes while improving their taste and nutritional quality to support dietary shifts.

Increasing accessibility and convenience of healthy, affordable plant-based options in grocery stores and restaurants in the U.S. would benefit those following a plant-based diet. Research has demonstrated that greater availability and visibility of plant-based products facilitates increased consumer usage [60]. Adequate food supply factors in the environment, such as access to supermarkets offering a variety of plant-based products, can help reduce meat consumption among meat-eaters [60, 61]. Additionally, some participants highlighted the need for taking supplements and the feeling of being unsatiated due to insufficient quality plant protein options when eating out. A systematic review of 48 studies found that while total protein intake among vegans was generally low, adequate levels could be achieved through the consumption of protein-dense plant foods such as legumes, nuts, meat analogs, and soya protein foods [62]. Vitamin B_12_ deficiency was commonly reported among vegans, but among those who consumed B_12_-fortified foods, the deficiency rate was 0% [63]. These findings, along with participants’ perceptions, underscored the importance of reformulating plant-based products, such as fortifying them with essential vitamins and offering more protein-dense options to meet diverse health and nutritional needs.

Other important facilitators included access to more recipes and support from people knowledgeable about plant-based diets. Evidence suggests that a lack of cooking skills and difficulties in finding the right recipes can hinder the progress from the intention to reduce meat consumption to actual behavior change [64, 65]. A qualitative study conducted in Quebec, Canada also emphasized the importance of skill development in meal preparation and food acquisition for adopting a whole-food plant-based eating pattern, as participants felt that a 12-week nutritional education program was insufficient to build these skills [66]. Additionally, individual cooking skills and recipe knowledge are predictive of the amount of food waste [67]. Hence, future interventions aimed at reducing meat consumption should focus on offering alternative food options and providing sufficient resources (e.g., recipes and cooking classes) to enhance individuals’ confidence to consume plant-based diets.

Strengths of this study include the use of a qualitative research methodology, which allowed for a deeper exploration of perceptions about consuming a plant-based diet within the current U.S. food system. Furthermore, the application of reflexive thematic analysis enhanced the validity. A trained nutrition researcher and registered dietitian conducted open-ended, semi-structured interviews to gather true perspectives in a comfortable setting. Transcripts were verified for accuracy, codes were independently developed by two researchers to minimize bias, and themes were collaboratively refined to strengthen thematic robustness through diverse perspectives.

Yet, several limitations of this study should be acknowledged. First, the sample size is relatively small, which primarily comprises White and Asian adults from Baltimore-area vegetarian and vegan communities, so findings on perceptions of adopting plant-based diets are not generalizable to individuals from diverse ethnic backgrounds, those living in rural and remote areas, and omnivores who are interested in consuming plant-based diets. Nonetheless, these findings are particularly informative for the design of future trials targeted at this study population. Although the inclusion criteria allowed for individuals interested in adopting a plant-based diet, all participants were already following vegan, vegetarian, or flexitarian diets. As a result, this study did not capture perspectives of omnivores considering dietary change. Additionally, as we did not collect data on participants’ socioeconomic status, we are unable to assess how socioeconomic differences may have influenced perspectives and behaviors. Selection bias may also be present, as those without Internet access or availability for in-person interviews might have different experiences with plant-based diets. Additionally, despite the research team reviewing and agreeing on the codes and thematic analysis, the interpretation of qualitative data remains inherently subjective. Future studies should use triangulation to enhance the credibility of the findings.

The findings of this study elucidated the perceived motivators and barriers to consuming a plant-based diet among U.S. adults and explored key factors relevant to dietary adherence. Availability of high-quality, affordable plant-based options and meat alternatives, social and familial support, and sufficient nutritional information and education on food preparation and purchasing are important determinants of following a plant-based diet. Future intervention studies should incorporate these insights to improve recruitment and engagement. Public health strategies could also address these motivators and barriers to promote the consumption of plant-based diets for the broader U.S. population for both human health as well as environmental sustainability.

## Electronic supplementary material

Below is the link to the electronic supplementary material.

Supplementary Material 1

## Data Availability

Data will be provided upon request to the corresponding author.

## Footnotes

1 The figure was developed by authors based on answers from interview questions: What are the advantages/disadvantages of consuming plant-based diet? What are some health/environmental benefits you would think of when consuming a plant-based diet? Do you think that consuming a plant-based diet will help with your weight management (loss/maintenance) and will this be a factor influence your adherence? What are some concerns you would think of when consuming a plant-based diet? These concerns can be related to health, social factors, of the environment. Do you have concerns related to health, social factors, cooking, accessibility, taste or price? What kind of barriers may hinder the adoption of a plant-based diet?

2 Color shading was used to group phrases that shared similar semantic content or reflected contrasting perceptions across participants. Phrases left unshaded (white) were not intended to be thematically grouped and represent standalone concepts

## Abbreviations

EAT Plants 1 study: Eating, Acceptability, and Taste of Plants 1 study
TPB: Theory of Planned Behavior
