# Supplementary material for: Perceived motivators and barriers to consuming a plant-based diet: a qualitative research study
Source: BMC Nutr. 2025 Jun 2;11:108. doi: 10.1186/s40795-025-01100-7 (PMC12131402; doi:10.1186/s40795-025-01100-7)
Supplement: Supplementary file 1 — Supplementary Material 1 [file 40795_2025_1100_MOESM1_ESM.docx]

**Appendix 1.** Semi-structured interview guide based on Theory of Planned Behavior

Attitude (behavioral beliefs)

- What are the advantages/disadvantages of consuming plant-based diet?
- What are some health/environmental benefits you would think of when consuming a plant-based diet?
- Do you think that consuming a plant-based diet will help with your weight management (loss/maintenance) and will this be a factor influence your adherence?
- What are some concerns you would think of when consuming a plant-based diet? These concerns can be related to health, social factors, of the environment.

Social norms (normative beliefs)

- Who are the important people (family member or friends) for you who would approve/disapprove of your consumption of plant-based diet?
- Approval/disagreement may be shown through what ways? How might their approval or disapproval be shown?
- Will you be influenced by the opinions from others?

Perceived behavioral control (control beliefs)

- What would make it easier for you to consume plant-based diet?
- What kind of barriers may hinder the adoption of a plant-based diet?
- Do you have concerns related to health, social factors, cooking, accessibility, taste or price?
- Would the nutritional composition of processed foods be a concern when consuming a plant-based diet for you?
- What are your thoughts on consuming meat and dairy alternatives?
